# Mucilage extracted from Chilean papaya seeds is enriched with homogalacturonan domains

**DOI:** 10.3389/fpls.2024.1380533

**Published:** 2024-05-30

**Authors:** Dayan Sanhueza, Pablo Sepúlveda-Orellana, Alejandra Salazar-Carrasco, Sebastian Zúñiga, Raúl Herrera, María Alejandra Moya-León, Susana Saez-Aguayo

**Affiliations:** ^1^ Centro de Biotecnología Vegetal, Laboratorio Mucilab, Facultad de Ciencias de la Vida, Universidad Andrés Bello, Santiago, Chile; ^2^ Agencia Nacional de Investigación y Desarollo (ANID) - Anillo de Investigación en Ciencia y Tecnología - Chilean Fruits Cell Wall Components as Biotechnological Resources (CHICOBIO) ACT210025, Talca, Chile; ^3^ Laboratorio de Fisiología Vegetal y Genética Molecular, Instituto de Ciencias Biológicas, Universidad de Talca, Talca, Chile; ^4^ Agencia Nacional de Investigación y Desarollo (ANID) - Millennium Science Initiative Program - Millennium Nucleus for the Development of Super Adaptable Plants (MN-SAP), Santiago, Chile

**Keywords:** pectin, methylesterification, polysaccharides, hemicellulose, seed mucilage

## Abstract

Chilean papaya, also known as mountain papaya (*Vasconcellea pubescens*), is a fruit valued for its nutritional value and pleasant fragrance. The oblong fruit, featuring five ridges and a seed-filled mucilage cavity, is typically consumed cooked due to its high protease content. The mucilage and the seeds are usually discarded as byproducts. This study analyzed the biochemical composition of mountain papaya seed mucilage using methods such as HPAEC and immunolabeling. Results revealed that papaya seeds yield nearly 20% of their weight in mucilage polysaccharides, which can be separated into soluble and adherent layers. The mucilage exhibited a high proportion of acidic sugars, indicating that homogalacturonan (HG) is the predominant domain. It also contained other domains like rhamnogalacturonan-I (RG-I) and hemicelluloses, predominantly xyloglucan. The HG-rich mucilage, currently considered waste, emerges as a promising source of polysaccharides, indicating its multifaceted utility in various industrial applications.

## 1 Introduction

Chilean papaya fruit (*Vasconcellea pubescens*), also known as mountain papaya, is the local papaya grown in Chile. Because the plant is sensitive to cold temperatures, it is primarily cultivated in the north of Chile (Coquimbo region) and in the southern coastal areas that are protected from cold (Lipimávida, Del Maule region). The fruit is oblong with five pronounced ridges and contains an interior cavity filled with seeds embedded in a mucilage matrix (Gaete-Eastman et al., 2009; Letelier et al., 2020). During ripening, the fruit turns yellow and develops a strong and characteristic fruity aroma (Moya-León et al., 2004). It is a rich source of vitamins, including vitamins C and A, as well as, potassium, sugar, fiber and antioxidants (Vega-Gálvez et al., 2021). Chilean papaya is typically harvested when fully ripe and is commonly consumed in cooked forms, such as preserved fruit, juice, jam, or sweets (Vega-Gálvez et al., 2021). Unfortunately, because of this processing, papaya mucilage and seeds are often discarded as waste.

Plant mucilages are jelly-like structures produced by several plants, such as aloe vera, flax or chia seeds, and they are largely used by the industry due to their rheological properties (Eshun and He, 2004; Dybka-Stępién et al., 2021; Tosif et al., 2021; Poligundla and Lim, 2022). In the food industry, mucilage serves as thickener, emulsifier, and stabilizer in products like jellies, jams, and sauces. In the pharmaceutical sector, mucilage acts as an excipient or forms bio-capsules in the production of medicines owing to its gel-forming capacity and ability to provide a protective coating to the digestive tract. Mucilages also feature prominently in cosmetics as moisturizing agents in lotions and creams, among other products. Additionally, they play a role in the paper and textile industry as binders and improving the final product’s appearance (Tosif et al., 2021).

Despite their widespread use, there is limited information available about the chemical composition of plant mucilages. Among the better-studied mucilages are those from flax and Arabidopsis seeds. Both are primarily composed of rhamnogalacturonan-I (RG-I) pectic domains with varying proportions of hemicelluloses (HC) (Macquet et al., 2007; Naran et al., 2008; Kamel et al., 2020). RG-I consists of a backbone of rhamnose (Rha) and galacturonic acid (GalA), which can be branched with galactan, arabinan and or arabinogalactan side chains (Mohnen, 2008; Kaczmarska et al., 2022). Flax seed mucilage contains substantial amounts of arabinoxylan, with a minor presence of RG-I branched with galactan and arabinogalactan (Elboutachfaiti et al., 2017). In contrast, the well-characterized Arabidopsis seed mucilage is primarily composed of unsubstituted RG-I, with traces of xylan, galactoglucomannan and xyloglucan hemicelluloses domains (Macquet et al., 2007; Voiniciuc et al., 2015a). Arabidopsis mucilage also contains approximately 10% of homogalacturonan (HG) pectic domains, which correspond to a backbone of GalA that can be methylesterified, significantly influencing mucilage density and release (Saez-Aguayo et al., 2013; Parra-Rojas et al., 2023). Other mucilages have also been characterized as for example *Plantago ovata*, which is primarily composed of xylan (Phan et al., 2020). Tamarind seeds contain a heteropolysaccharide consisting of glucose (Glc), xylose (Xyl), and galactose (Gal) (Khanna et al., 1987), with chemical composition comprising approximately 86.2% neutral sugar and 5.4% uronic acid (Shao et al., 2019). Lastly, chia mucilage is composed of a novel, unidentified polysaccharide mainly comprising Xyl, Glc and 4-O-methyl-α-D-glucuronic acid as its primary components (Lin et al., 1994; Salgado-Cruz et al., 2013). These studies have shown that mucilage exhibits a heterogeneous composition that can vary depending on the analyzed species. So far, the main mucilaginous polysaccharide identified include RG-I, xylan, cellulose, and a small amount of HG. As there is no available information regarding the composition of mountain papaya seed mucilage, this study aimed to conduct biochemical and cytological analyses to uncover its composition.

This investigation highlights the characterization of a new mucilage derived from papaya seeds, which represent a novel and exploitable source of mucilage polysaccharides. Our analyses revealed a distinctive composition of papaya mucilage, primarily comprising GalA, a key component of homogalacturonan (HG), with a notable level of methyl esterification. Biochemical and cytological experiments provided evidence for the organized composition of this novel mucilage. These findings offer promise for utilizing papaya fruit waste, which is often overlooked by the industry.

## 2 Materials and methods

### 2.1 Plant material

Seeds were collected from the internal cavity of mountain papaya fruits harvested at Lipimávida (coastal area of Del Maule region, Chile). Seeds were separated, washed with water and then allowed to air-dry.

### 2.2 Ruthenium red staining

Two groups of dry papaya seeds were incubated in water for 3 and 24 h, respectively, and then stained with Ruthenium red solution (0.02% w/v) to visualize the mucilage. For CaCl_2_ (0.3 M) and EDTA (0.3 M) treatments, dry seeds were incubated for 3 h, rinsed twice with distilled water and then stained with Ruthenium red at 0.02% (w/v).

### 2.3 Papaya mucilage extraction and AIR preparations

For sequential extractions of mucilage, approximately 6 units (aprox. 60 mg) of dried papaya seeds were incubated in 5 mL of distilled water for 4 h on a rotary mixer at room temperature (RT). Supernatants were collected and named as soluble mucilage (SM). Seeds with adherent mucilage (AM) were sonicated in 5 mL of water for 6 min at 40% power using a sonicator (SONIC RUPTOR 250, OMNI INTERNATIONAL). Seeds were rinsed two times with 5 mL of water and the three fractions were pooled as AM. For total mucilage extraction, the same amount of seeds were embedded in 5 mL of water for 24 h and the mucilage was separated manually from the seeds using pair of tweezers.

Mucilage fractions were boiled for 5 min and then lyophilized. Then, dried mucilages were ground to form a homogenous powder with the help of liquid nitrogen and alcohol insoluble residues (AIR) were prepared by incubating the mucilage with 80% (v/v) aqueous ethanol overnight. The precipitate was washed twice using 1:1 (v/v) methanol:chloroform for 2 h each, followed by three washes with acetone for 45 min each. In each step, the liquid was removed by centrifugation at 4,400 g for 5 min, to precipitate the AIR, and fresh solution was added. The samples were then dried overnight at RT.

### 2.4 Papaya seed mucilage AIR fractionation, acid hydrolysis, and sugar analysis

#### 2.4.1 Papaya seed mucilage AIR fractionation

AIR fractions generated from extracted soluble and adherent papaya seed mucilage (as described in 2.3) were used to isolate separate fractions of pectins and hemicelluloses. To isolate pectins the AIR was incubated with imidazole (0.5 M, pH 7.0) at 28 °C for 5 h on a rotary mixer. After centrifugation at 4,400 x g, for 5 min, the supernatant was recovered, and the pectin extraction continued with ammonium oxalate solution (0.2 M, pH 4.3) for 15 h at 28 °C. This procedure was repeated three times to thoroughly extract pectins. Imidazole and ammonium oxalate extracts were dialysed against water using a 10 kDa MWCO membrane (SnakeSkin™, ThermoFisher). After dialysis the fractions were combined to get one single pectin fraction. To obtain hemicelluloses the remaining AIR residue, after pectin extraction, was rinsed with water and incubated overnight with NaOH/NaBH_4_ (6M; 1% w/v) at 37 °C on a shaker. This incubation was repeated one more time. Supernatants were mixed and dialysed against water using a 10 kDa MWCO membrane. After dialysis the fractions were freeze dried.

#### 2.4.2 Acid hydrolysis

To analyze the monosaccharide composition in the different fractions the samples were hydrolyzed with 2 M trifluoroacetic acid (TFA) for 1 h (AIR), 45 min (pectin and HC) or 30 min (pure RG-I and OGAs) at 121 °C, using allose and myo-inositol (250 µM each) as internal standards. Samples were dried at 45 °C with gaseous nitrogen, rinsed twice with isopropanol and dried as previously described. The pellet was resuspended in 1 mL distilled water, sonicated for 15 min and filtered (pore size: 0.22 µm filter).

#### 2.4.3 Sugar analysis by HPAEC-PAD

The monosaccharide composition analysis and quantification were carried out through HPAEC-PAD using a ionex ICS-3000 HPAEC-PAD equipped with a CarboPac PA1 (4×250 mm) analytical column, and a CarboPac PA1 (4×50 mm) guard column. The separation of neutral sugars was performed at 32°C with a flow rate of 1 mL min^–1^ using an isocratic gradient of 20 mM NaOH for 25 min, followed by a separation of acidic sugars using a mixture of 75 mM sodium acetate and 150 mM NaOH for 15 min at a flow rate of 1 mL min^–1^ at 32°C, followed by a wash with 200 mM NaOH for 5 min. After every run, the column was equilibrated with 20 mM NaOH for 6 min. Standard curves of neutral sugars (Fucose (Fuc), Rha, Ara, Gal, Glc, Mannose (Man) and Xyl) or acidic sugars (GalA and glucuronic acid (GlcA)) were used for quantification in a range from 3.125 to 200 µM.

### 2.5 Determination of methyl-esterification degree

The degree of methylesterification (DM) was analyzed in AIR preparations, as described by Anthon and Barrett (2004). Around 1-2 mg of AIR was resuspended in 50 µL of ultrapure water and saponified with 50 µL of NaOH (1 M) at 4 °C on ice for 1 h. The saponification was stopped by adding 50 µL of HCl (1 M) and then was added 600 µL of ultrapure water. A volume of 50 µL of saponified AIR solution was incubated with 100 µL of Tris-HCl (200 mM, pH 7.5), 40 µL of 3-methyl-2-benzothiazolinone hydrazone (MBTH, 3 mg mL^−1^) and 20 µL of alcohol oxidase from *Pichia pastoris* at 0.02 U µL^−1^ (Sigma A-2404) and incubated for 20 min at 30 °C. The color was developed by adding to the mix 200 µL of sulfamic acid and ammonium ferric sulfate dodecahydrate (0.5% w/v each in water) solution, the samples were incubated at RT for 20 min. Then 600 µL of ultrapure water were added and absorbance at 620 nm was recorded. Methanol contents were determined using a standard curve between 0 and 10 ug µL^−1^ of methanol. All experiments were done using five technical replicates and three independent AIR preparations per sample.

### 2.6 Uronic acids quantification

The content of uronic acids in pectin fractions collected from SEC was analyzed using the m-hydroxybiphenyl method, as described by Blumenkrantz and Asboe-Hansen (1973). A volume of 2 µL out of a 3 mg mL^-1^ pectin solution was mixed with 18 µL of water and 100 µL of a 0.5% (w/v) solution of N_2_B_4_O_7_ x 10 H_2_O (borax) in sulfuric acid. The samples were incubated at 100°C for 5 min, then cooled and absorbance was recorded at 520 nm. To develop color, 2 µL of 0.15% (w/v) m-hydroxybiphenyl in 1 M NaOH solution was added, followed by a gentle mixing. The samples were allowed to stand for 5 min at RT before measuring the absorbance at 520 nm. The uronic acid content was determined using a standard curve based on 0.1 to 2 ug of GalA. All experiments were performed using six technical replicates and three independent AIR preparations per sample.

### 2.7 Pectin domains isolation

Material enriched in the different pectin domains (HG, RG-I and RG-II), was obtained by size exclusion chromatography (SEC) of endopolygalacturonase-treated isolated pectin. Firstly, the pectin was saponified by an overnight incubation with 0.2 M NaOH at 4 °C, then neutralized with 0.2 M HCl. The saponified pectin was precipitated with 2 volumes of ethanol, centrifuged for 2 min at 4,400 x g, and rinsed twice with 80% (v/v) aqueous ethanol. Next, the saponified pectin was digested overnight at 20 °C with 2.25 U mL^−1^ of endo-polygalacturonase from *Aspergillus aculeatus* (Megazyme) in pyridine:acetic acid:water (PyAW 1:10:200, v:v:v) solution. The digestion was loaded into a Bio-Gel P-30 column (2.5 x 57 cm) and eluted with PyAW 1:1:98 at 1 mL min^-1^. Fractions of 5 mL were collected, and 200 µL from each fraction were used to quantify total uronic acids. Fractions enriched in RG-I, RG-II, and OGAs were separately pooled, dried in a speed vacuum, and finally resuspended in 1 mL water for further analysis.

### 2.8 Immunodot blot assay

Immuno-dot blot assays were performed using fractions of pectins and hemicelluloses from papaya seed mucilage, as well as, a purified fraction of RG-I obtained from size exclusion chromatography (SEC) as previously described. The primary antibodies used are described in **Supplementary Table 1**. A constant volume of 0.7 µL, using a series of dilutions starting from 2 µg µL^-1^ down to 0.25 µg µL^-1^ of either fraction, was blotted onto a 0.45 µm nitrocellulose membrane (Thermo Scientific, cat. No. 88018). The blotted membranes were incubated with a blocking solution containing 3% (w/v) of powdered skim milk dissolved in TBS (25 mM Tris, 0.15 M NaCl) supplemented with 0.1% (v/v) of Tween-20 (TBS-T). All primary antibodies were diluted to 1:50 in TBS-T with 1% (w/v) skim milk, and the membranes were incubated for 2 h at RT, except for the 2F4 antibody, which was diluted to 1:50 in 20 mM Tris-HCl pH 8.2, 0.5 mM CaCl_2_, 150 mM NaCl (TcaNa buffer according to the manufacturer’s recommendation) supplemented with 1% (w/v) skim milk and 0.1% (v/v) Tween-20.

After incubation with the primary antibody, the membranes were washed three times with TBS and incubated for 1.5 h at RT with the corresponding secondary antibody diluted to 1: 2,000 in TBS-T. Finally, the membranes were washed and incubated with 1 Step BCIP/NBT (5-bromo-4-chloro-3-indolyl-phosphate/nitro blue tetrazolium) chromogenic alkaline phosphatase (AP) substrate developing solution (Thermo Scientific, cat. No. 34042). The reaction was stopped by washing the membranes three times with distilled water. The dot signals were semi-quantified using ImageJ 1.53 software (Freeware, National Institute of Health). A minimum of three technical replicates were used for the immunoblot analysis of each pectin and hemicellulose fraction.

### 2.9 Histological structure analysis and immunolabeling

Histological sections were prepared using dehydrated and, then hydrated papaya seeds for 3 h in distilled water. The resin imbibition starts with samples (seeds) fixation in FAA solution (10% formaldehyde, 5% acetic acid, 50% ethanol, and 35% distilled water) followed by seed dehydration, involving sequential 1 h incubations in ascending ethanol concentrations (30%, 50%, 70%, 80%, 95%, and 100%). Subsequently, the seeds were embedded in a series of three ethanol/LR White resin steps, concluding with 100% resin (Ted Pella). Sections 1 µm thick were cut using a Leica EM UC7 ultramicrotome (Leica Biosystems, Wetzlar, Germany), stained with 0.1% (w/v) toluidine blue in 0.1 M phosphate buffer at pH 6.8, and analyzed using a Leica DM500 light microscope with a Leica ICC50 HD camera.

The immunofluorescence labeling was performed using six monoclonal antibodies that recognize HG, RG-I, galactan, and AGP epitopes. The JIM5, JIM7, and 2F4 antibodies were employed to detect HG in different structures and degrees of methylesterification, as each antibody recognizes HG with a low degree of methylesterification, highly-methylesterified HG, and egg-box structures, respectively. The INRA-RU1 antibody was used to label the RG-I backbone. The LM26 antibody recognizes galactosyl substitution in galactan branches of RG-I, and the LM30 antibody recognizes the carbohydrate epitope containing β-linked glucuronic acid in the arabinogalactan protein (Willats et al., 2000; Verhertbruggen et al., 2009; Ralet et al., 2010; Saez-Aguayo et al., 2013, 2017). To stain the tissue surface Calcofluor White (0.01% w/v) was used. Images from optical sections were obtained using a Leica TCS LSI spectral confocal laser scanning microscope. A 488 nm argon laser line was used to excite Alexa Fluor 488, and a 405 nm diode laser line was used to excite Calcofluor White. Fluorescence emission was detected between 505–550 nm for Alexa Fluor 488 and between 412–490 nm for Calcofluor White.

## 3 Results

### 3.1 Papaya seed is surrounded by a large amount of lightly-attached mucilage gel-like matrix

**Figure 1:**
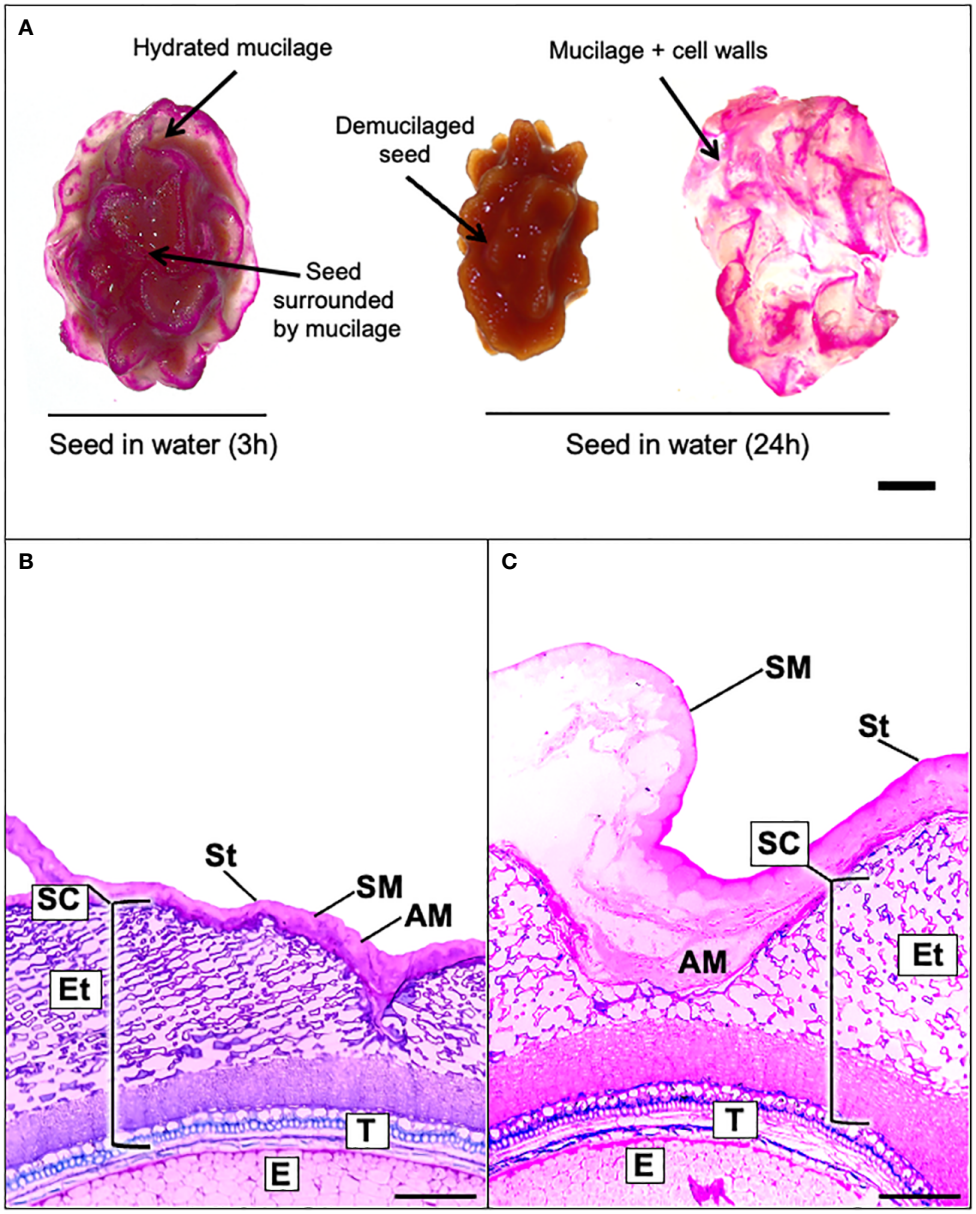
Papaya seed is surrounded by a mucilage capsule composed by a soluble and adherent layer. **(A)**, Mountain papaya seeds were incubated in 0.02% Ruthenium red solution after being soaked in distilled water for 3 h or 24 h. The papaya seed mucilage can easily be detached from the seed after soaking in water for 24 h. Scale bar = 5 mm. **(B, C)**. Light microscopy image of mature seed coat papaya section stained with toluidine blue. **(B)** Section of dehydrated seeds with compacted mucilage and **(C)** a 3 h hydrated seed with expanded imbibed mucilage. AM, Adherent mucilage; SM, soluble mucilage; St, β-glucan sarcotesta; SC, seed coat; Et, endotesta; T, tegument; E, endosperm. Scale bars = 200 µm. With imbibition in water, the separation of the adherent and soluble mucilage can be observed.

Papaya fruit contains many seeds whose surface is not smooth as in Arabidopsis, on the contrary, it is structured in the form of spikes surrounded by a whitish jelly-like matrix corresponding to seed mucilage (**Figure 1**). As a first approach, to attempt an initial identification of the polysaccharides that compose the papaya mucilage, a Ruthenium red staining was carried out after 3 h and 24 h of seed imbibition in water (**Figure 1**). Ruthenium red is a molecule which links to free carboxyl groups of GalA sugars, thereby staining acidic polysaccharides such as HG and RG-I domains in pink (Murano et al., 1990). After 3 h of imbibition, the mucilage looks like an irregular bag filled with a matrix that surrounds the seed (**Figure 1A**). This mucilage bag is hard, and it is not easy to remove with tweezers or detach from the seed surface by shaking. The pink stain of papaya mucilage suggests that its composition is mainly pectin. Interestingly, the Ruthenium red labeling is not regular as part of the mucilage surface stains as pink lines suggesting that these structures contain pectin, meanwhile the other part of the mucilage could be composed of neutral polysaccharides such as cellulose or HC that do not react with the stain. After 24 h of imbibition, the Ruthenium red pattern is the same, but the mucilage bag can be easily detached from the seed surface with tweezers suggesting that the adherence of papaya mucilage is not as strong as in Arabidopsis or flax seed mucilage (Macquet et al., 2007; Zhao et al., 2017). To obtain information about papaya mucilage structure, sections of seeds were obtained from both dry and water-soaked seeds and were stained with toluidine blue (**Figures 1B, C**). Papaya seed coat is composed of a tegument and an endotesta. The mucilage is strongly compacted in a bag that surrounds the seed coat, and no columellae are observed (**Figure 1B**). Upon imbibition in water, the tegument changes its structure and expands its cells, while the endotesta appears completely disintegrated and expanded. The mucilage is disrupted in some points, and the separation of a layer called the soluble mucilage (SM) from the adherent like mucilage (AM) is observed (**Figure 1C**).

### 3.2 The papaya seed mucilage is mainly composed of acidic sugars

**Figure 2:**
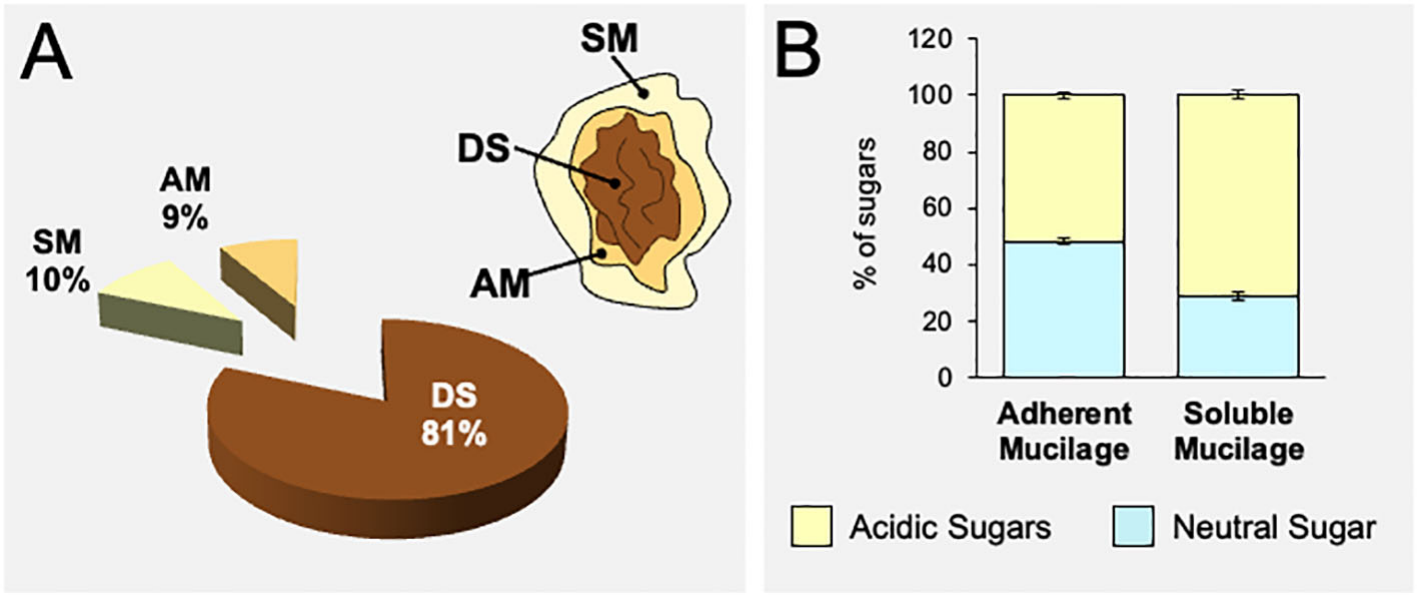
Papaya seeds produce a high amount of mucilage that is mainly composed of acidic sugars. **(A)** Quantification of adherent and soluble mucilage fractions separated from papaya seeds (n=12). Values are expressed based on weight. **(B)** Quantification of acidic and neutral sugars in the adherent and soluble mucilage fractions obtained from papaya seeds determined by HPAEC-PAD after TFA hydrolysis. Values correspond to mean ± standard error (SE) (n=12). AM, adherent mucilage; SM, soluble mucilage; DS, demucilaged seed. The acidic and neutral sugar composition of total AIR extracted from each mucilage layer is presented in **Supplementary Figure 2**.

Papaya seeds release a large capsule of polysaccharides when they are soaked in water. To estimate the percentage of mucilage produced, seeds were soaked in water for 24 h, then the mucilage was separated manually, freeze-dried, and weighed (**Supplementary Figure 1**). Results shown in **Figure 2A** indicate that approximately 19% of the papaya seed composition corresponds to mucilage, representing a significantly higher amount of polysaccharides compared to Arabidopsis that synthesizes only 3% of their weight in mucilage (Macquet et al., 2007; Voiniciuc et al., 2015a).

To delve deeper into the mucilage composition, we opted to conduct separate analyses of SM and AM layers. To accomplish this, the seeds were soaked in water for 4 h to extract the soluble mucilage. Following this, the seeds without the soluble mucilage underwent sonication to release the adherent mucilage (**Supplementary Figure 1**). The SM and AM fractions were used to prepare AIR extracts that were submitted to acidic hydrolysis to analyze its sugar composition. The monosaccharide analyses of each AIR layer show that both the soluble and adherent mucilages contain the same amount of total sugars, approximately 460 mg/g of AIR (**Supplementary Figure 2**). The analysis reveals that both layers of papaya mucilage are mainly composed of acidic sugars, mainly GalA (**Figure 2B**; **Supplementary Figure 2**). The main sugar contributing to both SM and AM layers is GalA, with 70.68% in the soluble layer and 50.36% in the adherent layer (**Supplementary Figure 2**). The following sugars in both the soluble and adherent layers are respectively Xyl (6.22% and 14.35%), Gal (6.13% and 9.72%), Rha (4.13% and 6.57%), and Ara (3.63% and 5.32%) (**Supplementary Figure 2**). Although similar sugar profiles are in general observed in both mucilage layers, the adherent mucilage contains more neutral sugars than the soluble mucilage (**Figure 2B**). These differences are mainly represented by a significant decrease in the amount of GalA measured in the adherent mucilage, while the levels of neutral sugars increased in a variable manner within the same mucilage layer. Due to the analysis being conducted on total AIR, it is not possible to conclusively determine whether the variations in neutral sugars are specifically associated with sugars belonging from the pectin or hemicellulose polysaccharides.

### 3.3 Homogalacturonans are key structures in the papaya mucilage for the adherence to the seed surface

**Figure 3:**
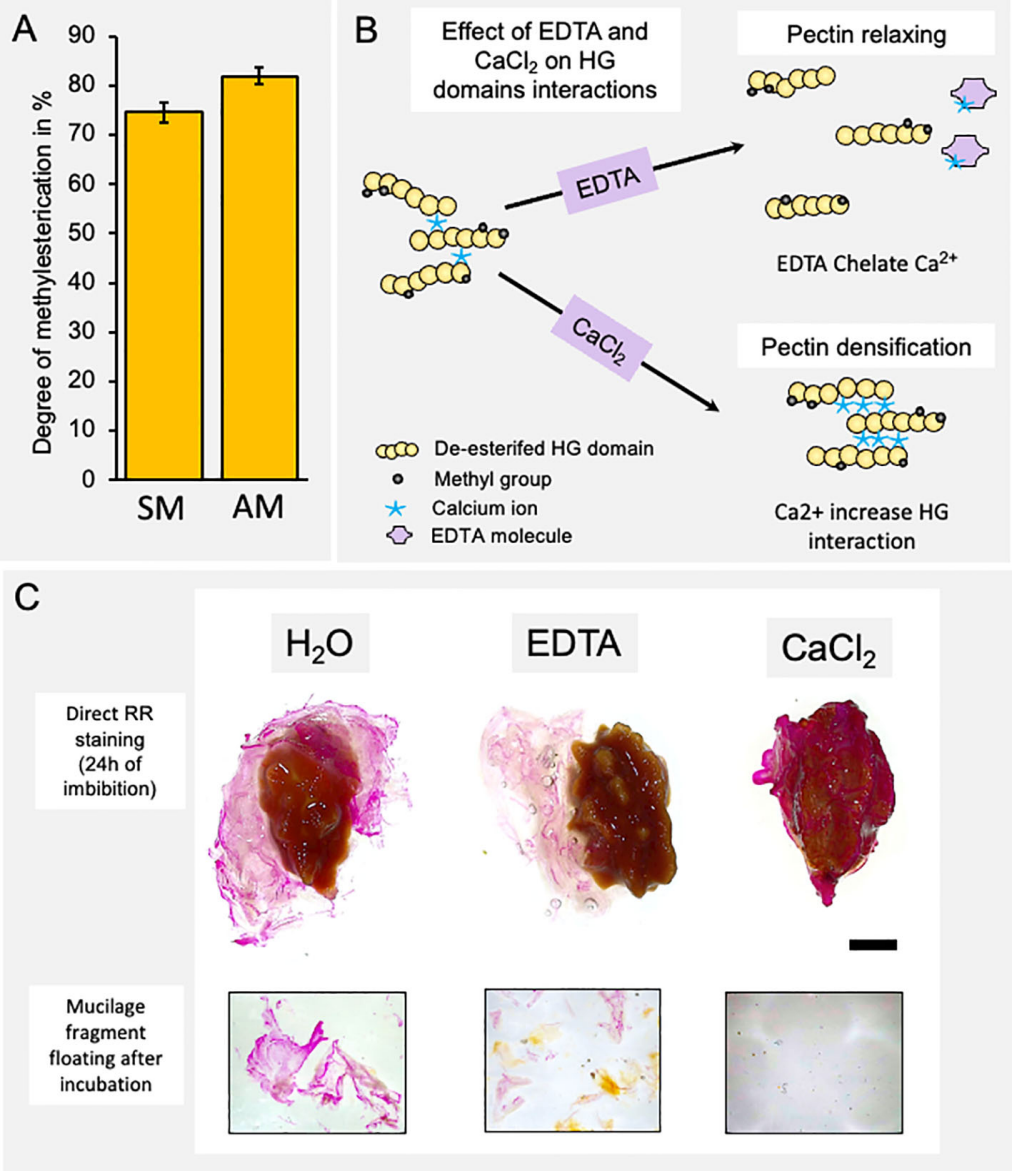
The homogalacturonan domain is a key molecule in the adherence of the mucilage to the seed surface. **(A)** Determination of the degree of methyl-esterification of each mucilage layer. Values correspond to mean ± SE from 10 technical replicates. **(B)** Schematic representation of the effect of a calcium chelator (EDTA) and calcium (CaCl_2_) on the homogalacturonan domain of pectins and on the rheological properties of pectins. **(C)** Papaya seed mucilage detachment under different soaking conditions: water, EDTA, or calcium (CaCl_2_). A view of the whole seed and a magnification of mucilage fragments released into the incubation medium. The presence of calcium ions strongly increases mucilage adherence. Scale Bar= 5mm.

The main monosaccharide component of papaya mucilage is GalA, suggesting that the HG domain is a relevant component in the mucilage structure (**Supplementary Figure 2**). In Arabidopsis mucilage, it has been reported that the level of methylesterification of GalA that forms HG is important for the structuration of Arabidopsis seed mucilage (Saez-Aguayo et al., 2013; Turbant et al., 2016; Parra-Rojas et al., 2023). Thus, to have an idea about the methylesterification degree of HG in papaya mucilage, a colorimetric assay was performed to determine the amount of methanol released after saponification. This methanol amount was then utilized to calculate the degree of methylesterification in papaya seed mucilage (**Figure 3A**). Results show a high degree of HG methylation in both mucilage layers (74.58% SM and 81.96% AM) suggesting that methylation participates in papaya seed mucilage structure. Thus, to determine the importance of HG domains and their methylation state in papaya mucilage adhesion, we tested the expansion of mucilage in presence of EDTA, a calcium chelator, and in the presence of calcium chloride (CaCl_2_) (scheme in **Figure 3B**). EDTA captures the Ca^2+^ ions and frees the egg-box domains formed between two demethylesterified HG, causing pectin relaxation, allowing more extensive hydration and swelling of mucilage and/or weakening of the primary cell wall (Arsovski et al., 2009; Ravn and Meyer, 2014). Contrarily, the addition of CaCl_2_ favors the polysaccharide matrix stiffness since it increases the demethylesterified HG domain to form egg-box structures. Following a 24 h period of water imbibition, the mucilage maintains a bag-like structure that encloses the seed (**Figure 3C**). During this process, a few large fragments are released into the surrounding water. In the presence of EDTA, which chelates calcium bridges between demethylated HG, the mucilage bag undergoes fragmentation, facilitating its separation from the seed surface. The detached mucilage is then fragmented into smaller pieces (**Figure 3C**). Lastly, when calcium is present, the mucilage remains dehydrated and adheres tightly to the seed surface, making it impossible to separate. No fragments of mucilage were observed in the imbibition solution. These results confirm the key role of HG in the adherence to the seed surface in papaya.

### 3.4 Homogalacturonan deposits in the papaya mucilage pocket are highly-organized

**Figure 4:**
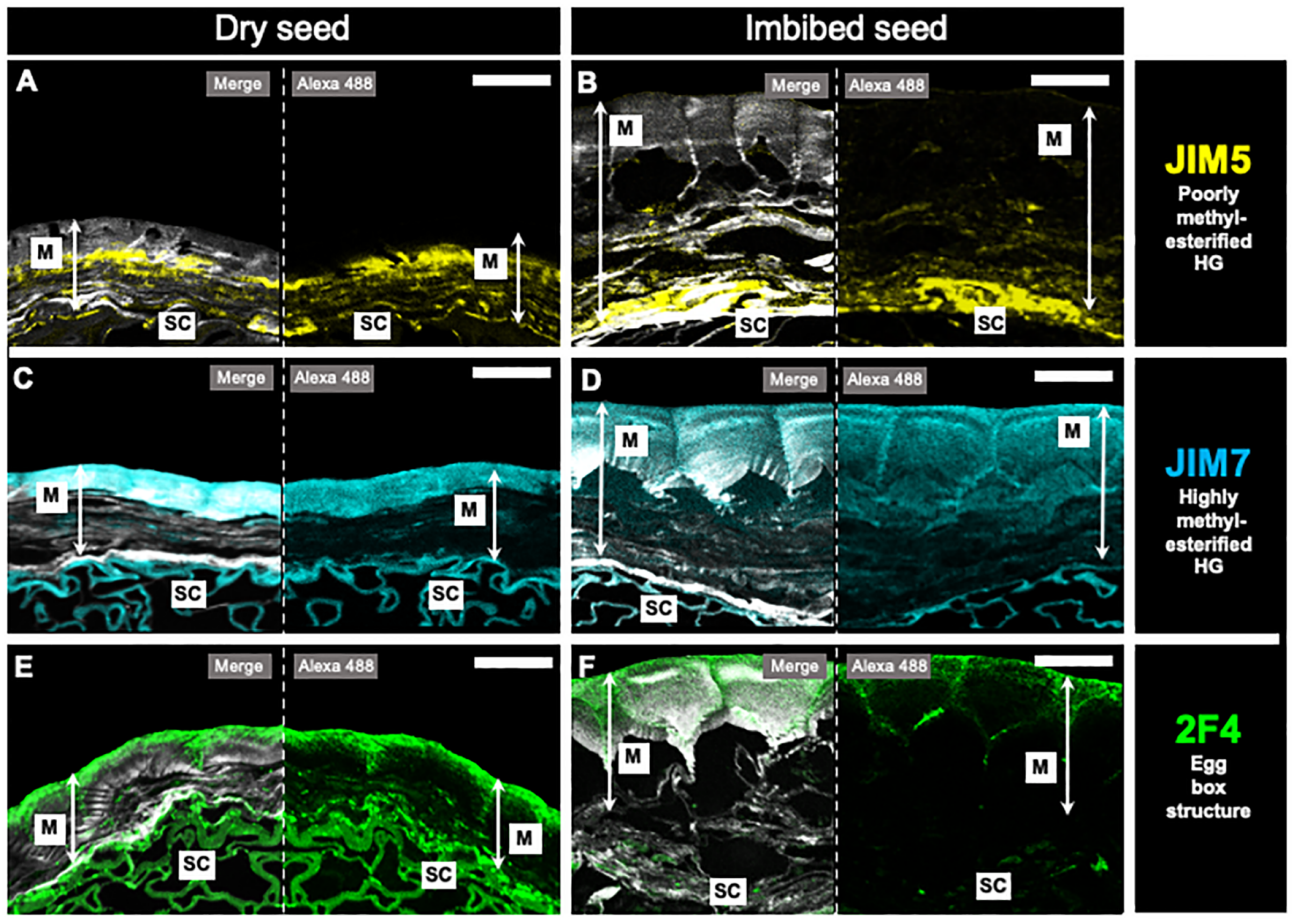
*In situ* homogalacturonan distribution in papaya mucilage determined by immunofluorescence. Immunofluorescence analyses were performed on dehydrated seeds **(A, C, E)** and water-soaked papaya mucilage **(B, D, F)** using the following antibodies: JIM5, labeling in yellow the poorly methyl-esterified HG domain **(A, B)**; JIM7, labeling in cyan the highly methyl-esterified HG domain **(C, D)**; 2F4, labeling in green the egg-box structures, and Calcofluor white staining for glycans and seed coat in gray **(E, F)**. SC, seed coat; M, mucilage. Scale bar = 30 µm.

To gain information about the structure and organization of HG in the papaya mucilage capsule, dry and imbibed seeds were fixed and embedded in LR white resin. Sections were then taken to label specific HG epitopes. The JIM5, JIM7, and 2F4 antibodies were used to identify poorly methylesterified HG, highly methylesterified HG, and egg-box structures formed by HG domains, respectively (**Figure 4**). In sections of dry seeds (**Figures 4A, C, E**), specific zones were labeled by the different antibodies. The JIM5 mark was localized near the seed surface, while the JIM7 signal was strongly accumulated in the distal area of the mucilage. The egg-box structures, identified by 2F4, were in the same zone as JIM7 and were more concentrated in the outermost edge of the distal wall of the papaya mucilage capsule (**Figure 4E**). When seeds were imbibed, the areas recognized by JIM7 and JIM5 remained (**Figures 4B, D**), but the antibody labeling for 2F4 was diffused throughout the mucilage, suggesting a diffusion or formation of micro gel egg- box structures upon imbibition (**Figure 4F**).

### 3.5 Adherent and soluble papaya mucilage have a similar composition and both are composed mainly of homogalacturonan domains

**Figure 5:**
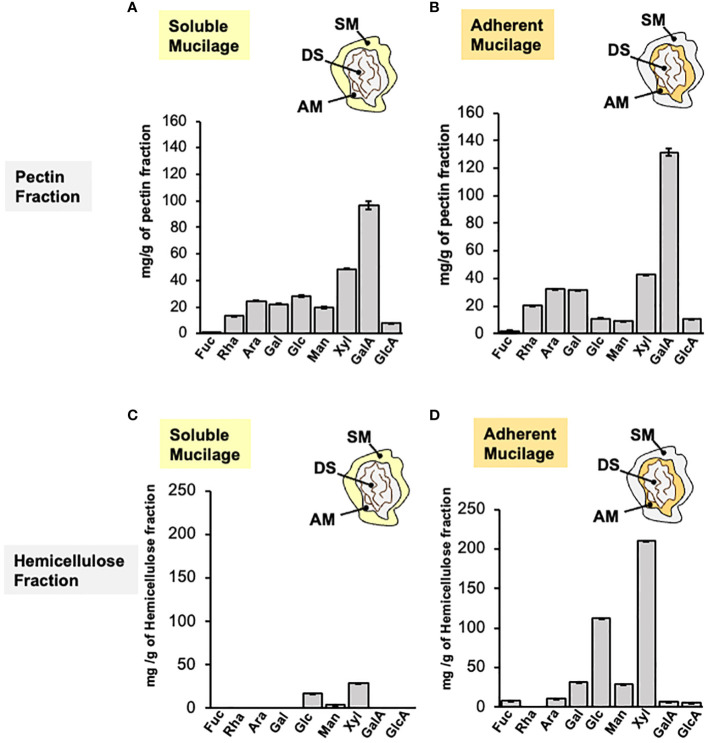
Monosaccharide composition of papaya mucilage layers after pectin and hemicellulose fractionation. Monosaccharide composition analysis of soluble **(A, C)** and adherent **(B, D)** papaya mucilages performed on pectin and hemicellulose fractions obtained by imidazole/ammonium oxalate and sodium hydroxide treatments, respectively. Sugar composition was determined by HPAEC-PAD after TFA hydrolysis (n=12). SM, soluble mucilage; AM, adherent mucilage; DS, demucilaged seed; Fuc, fucose; Rha, rhamnose; Ara, Arabinose; Gal, galactose; Glc, glucose; Man, mannose; Xyl, xylose; GalA, galacturonic acid; GlcA, glucuronic acid.

The monosaccharide composition analysis of AIR from SM and AM layers has uncovered a significant quantity of GalA associated with HG. However, it has also identified the presence of Rha, Gal, and Ara, which together comprise the RG-I domain. This RG-I domain could potentially play a role in the rheological properties of papaya mucilage. To provide more information about the pectin domains composing papaya mucilage, a fractionation of AIR was performed to separate pectin and hemicellulose domains in each layer (**Figure 5**). The pectin fractions of both mucilage layers share the same profile of monosaccharides, with a high amount of GalA that is around six times the Rha content, confirming the presence of HG as the main mucilage domain in the pectin fraction (**Figures 5A, B**). A significant amount of Xyl was also identified in SM as well as in AM, along with low amounts of other neutral sugars, suggesting that this fraction may contain a portion of HC and/or is part of the xylogalacturonan pectic domain. Finally, the presence of Rha, Gal, and Ara in the pectin fraction suggests the presence of branched RG-I.

The sugar profile in the HC fractions (**Figures 5C, D**) differs between the soluble and adherent layers. The HC fraction of the soluble layer contains only three sugars: Glc, Xyl, and Man, suggesting the presence of xyloglucan, xylan and/or glucomannan. The HC fraction of the adherent mucilage contains the same three neutral sugars, Xyl, Glc, and Man but in higher quantities. Additionally, it includes other neutral sugars such as Gal, Ara, and Fuc. This suggests the presence of the previously proposed domains for SM, along with galactoglucomannan, potential Ara substitution in xyloglucan, or possible remnants of the pectic fraction. To detect the HC epitopes in both mucilage layers, dot blot was carried out on isolated HC and we detect epitopes of xylan, xyloglucan and heteromannans on papaya mucilage (**Supplementary Figure 5**).

### 3.6 Pectic domain separation reveal the presence of rhamnogalacturonan-I, rhamnogalacturonan-II and xylogalacturonan in papaya seed mucilage

**Figure 6:**
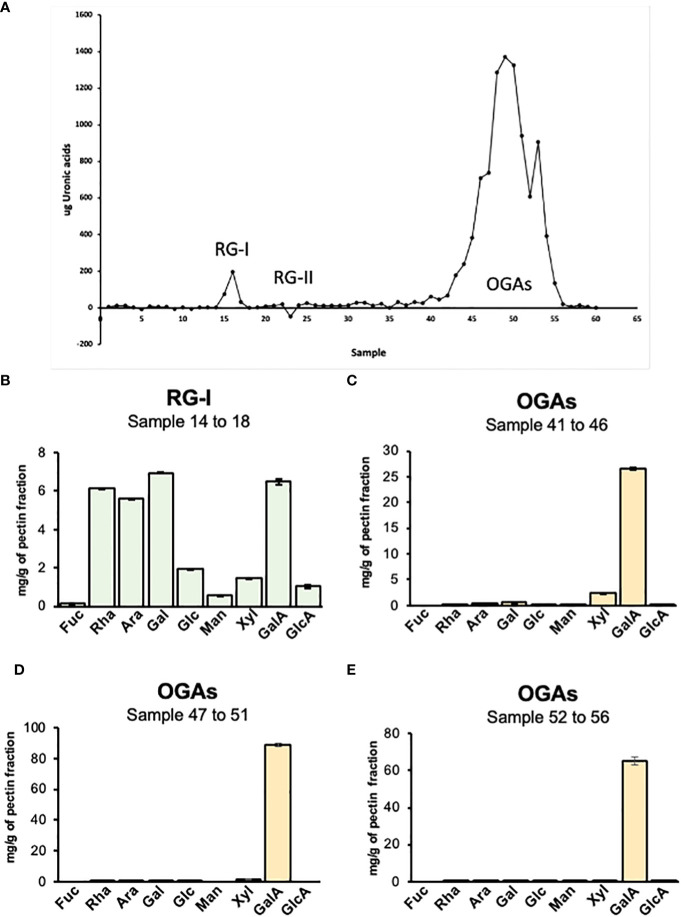
Analysis of pectin domains isolated by size exclusion chromatography. **(A)** Elution profile of pectin domains isolated by SEC. The graph indicates the peaks obtained by uronic acid quantification of each fraction collected from the Bio-Gel P-30 column. RG-I, fractions 14 to 18; RG-II, fractions 22 to 27; and OGAs, fractions 40 to 56. **(B–E)** Sugar profile for pectin isolated domains. The different graphs represent the result of the acidic hydrolysis assay performed to determine the specific composition of four pectin domains isolated through size exclusion chromatography. RG-I, rhamnogalacturonan-I; RG-II, rhamnogalacturonan-II; OGAs, oligogalacturonides.

To further investigate the specific pectin domains present in the mucilage, a preparation corresponding to pectin from total mucilage (SM+AM) was digested with endo-polygalacturonase (endoPG), which cleaves the HG domain without cutting RG-I or RG-II. The digestion product was loaded onto a chromatography column of Bio-Gel P-30 to separate it by size (Fry, 1988) (**Figure 6A**). By using the uronic acid quantification method, the elution profile was obtained, allowing us to specifically identify three peaks. The first peak, corresponding to RG-I, was small (fractions 14 to 18). The second peak (fractions 22 to 27), which was almost negligible, corresponded to RG-II (**Figure 6A**). Finally, a large peak (fractions 40 to 56) was observed, corresponding to oligogalacturonans (OGAs) produced by HG degradation. The main information obtained from this analysis confirms HG as the main pectin domain, with only a small amount of RG-I detected and the presence of traces of RG-II in the mucilaginous structure.

The different fractions obtained from the column were pooled based on their type and dried using a speed vac to obtain a single fraction corresponding to RG-I and RG-II. Since there was a high number of fractions corresponding to OGAs, they were pooled into three groups: 40 to 46, 47 to 51, and 52 to 56. The different samples were hydrolyzed with TFA to determine their sugar composition (**Figures 6B-E**). The fraction corresponding to RG-I presented a profile where GalA, Rha, Ara, and Gal were the principal sugars. The GalA/Rha ratio was 0.93, which is close to 1, the expected value for RG-I, considering that its backbone is composed of a disaccharide of GalA and Rha. The high content of Ara and Gal suggests a highly branched RG-I structure. The analysis of the three OGA fractions showed that these fractions are composed almost entirely of GalA, indicating that they are pure HG. A very low amount of Xyl was detected in the 40-46 pool and decreased in the other two pools. Since RG-II is a complex structure with six side branches and sugars that are not detectable by HPLC, the best method to specifically visualize and confirm the presence of this domain is by PAGE and silver staining. The gel exhibited a strong band corresponding to the dimeric form of this domain, indicating that almost, if not all, of the RG-II present in the cell wall is crosslinked with boron (**Supplementary Figure 3**).

### 3.7 Rhamnogalacturonan-I co-localized with AGPs and xyloglucan

Specific antibodies were employed to analyze the isolated RG-I domain, aiming to identify precise pectic epitopes to gain a deeper understanding of its structure (**Supplementary Figure 4**). To conduct this, the RG-I fraction were quantified based on their uronic acid content spotted with decreasing concentration. The most intense spots were observed for the highest concentration, which tended to decrease or completely disappear in diluted samples. INRA-RU1, which recognizes unbranched RG-I and requires at least 6 disaccharide backbone repeats for binding (Ralet et al., 2010), displayed a clear spot that was consistently observed across all dilutions, albeit not of very high intensity. INRA-RU2, which also recognizes unbranched RG-I showing significant binding to 2 disaccharide backbone repeats, but maximal binding to 7 disaccharide repeats (Ralet et al., 2010), exhibited a pattern similar to that observed for INRA-RU1. Another antibody showing a similar profile was LM6, which identifies a linear pentasaccharide in (1-5)-α-L-arabinans from the RG-I side chain (Willats et al., 1998). The labeling from this antibody aligned well with the monosaccharide composition analysis, revealing a high content of arabinose related to RG-I side chain. LM26, which recognizes branched 1,4-galactan, exhibited a faint signal that followed a pattern similar to LM6. The labeling corresponded with the observed levels of galactose quantification from isolated RG-I acid hydrolysis. Given that xyloglucan is one of the polysaccharides present in Arabidopsis mucilage (Voiniciuc et al., 2015a) and we observed a considerable amount of Xyl and Glc in our monosaccharide analysis, we employed LM25 (XXXG, XXLG, XLLG) to detect the presence of different HC epitopes (Pedersen et al., 2012). An intense label was observed across all tested dilutions, strongly indicating the presence of xyloglucan. To fully clarify the origin of xylose in the RG-I analysis, LM10 and LM11 antibodies were used to analyze the presence of xylan. Neither of them produced a label, indicating a clear absence of reaction, thus discarding the presence of xylan (Mc Cartney et al., 2005; Ruprecht et al., 2017). However, due to the intense label for LM25, it is impossible to rule out a potential contamination with HC. Lastly, considering recent data concerning the binding between RG-I and arabinogalactan proteins (AGPs), the LM30 antibody was included in the analysis; however, the labeling was weak, even at higher concentrations of RG-I (Knox et al., 1991; Moller et al., 2008).

**Figure 7:**
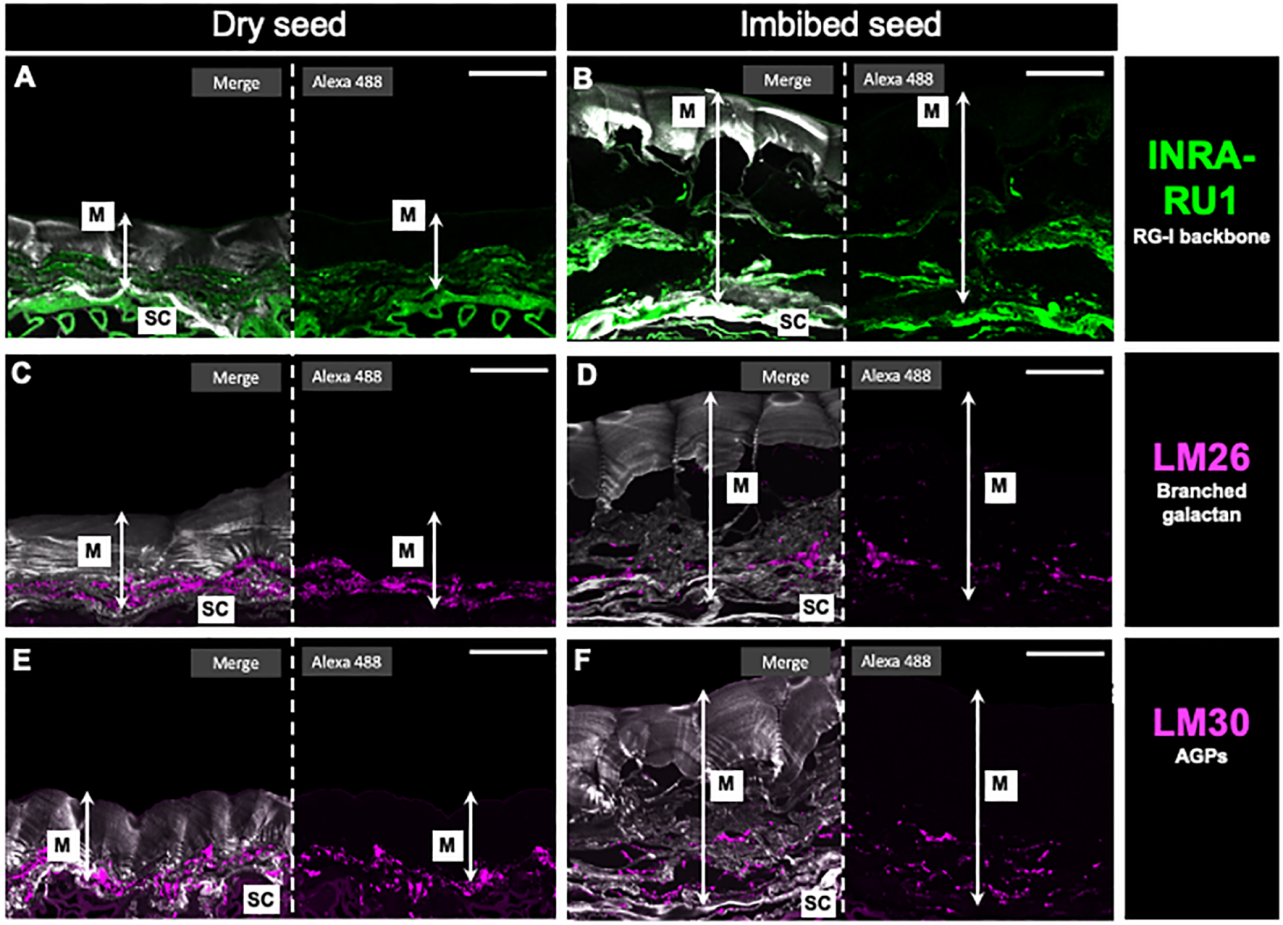
*In situ* rhamnogalacturonan-I distribution in papaya mucilage determined by immunofluorescence. Immunofluorescence analyses were performed on dehydrated seeds **(A, C, E)** and water-soaked papaya mucilage **(B, D, F)** using the following antibodies: INRA-RU1, labeling in green the unbranched RG-I backbone **(A, B)**; LM26, labeling in magenta the branched galactan in RG-I side chain **(C, D)**; LM30, labeling in magenta the arabinogalactan proteins, and Calcofluor white staining for glycans and seed coat in gray **(E, F)**. SC, seed coat; M, mucilage. Scale bar: 30 µm.

In addition to the dot blot analysis, an immunohistochemical assay was conducted on seed slices from both dry and imbibed seeds using the INRA-RU1 antibody (**Figures 7A, B**). In the dry seeds, the majority of the labeling was observed in the seed coat. In contrast, in the imbibed seeds, the labeling seemed to be more widespread, likely due to the release of mucilage and subsequent swelling. The labeling observed for LM26 in dry seeds (**Figure 7C**) appears concentrated in the region near the seed coat. However, upon hydration (**Figure 7D**), a portion of the label disappears, and it also appears disrupted, with some dots becoming visible further from the seed coat. The labeling observed for LM30, specifically for AGPs, displayed a similar pattern to what was observed with LM26. In the case of LM30, the label also had a structure near the seed coat that tended to diminish and spread out after imbibition (**Figures 7E, F**). Lastly, xyloglucans were labeled using LM25 antibody showing a uniform distribution throughout the entire mucilage without a distinct preference under organized stacked sheets (**Supplementary Figure 6**).

## 4 Discussion

The high polysaccharide content (approximately 19%) in papaya seeds’ mucilage, compared to species like Arabidopsis with 3-4%, positions papaya seeds as a valuable polysaccharide source (**Figure 2**). Varied quantities prompt inquiries into whether mucilage accumulation in seed coat epidermal cells could be constrained by space limitations, thereby decreasing polysaccharide production. This raises questions about mucilage-producing seeds in the pericarp, like tomatoes, and whether they yield larger quantities. Comparisons of different mucilages, as found in tomatoes, cucumbers, and passion fruit, could offer insights into their production, quantity, and composition, further enhancing our understanding of seed mucilage dynamics.

An additional question concerning the nature of the polysaccharide in papaya mucilage—is it akin to that in other species like Arabidopsis?. When we analyzed papaya mucilage structure with Ruthenium red, the pink stain indicates a predominantly pectin composition, binding to acidic polysaccharides and negatively charged groups. However, the non-uniform, patchy color distribution (**Figure 1**) differs from Arabidopsis seed mucilage, potentially tied to high methylation in both SM and AM layers or localized deposition of HG and HC within the mucilage matrix. This irregular pattern highlights particular papaya mucilage structure is not observed in Arabidopsis.

Although most of the homogalacturonan (HG) in the cell wall appears methylated and localized in the whole mucilage bag, they are mainly unavailable for dye binding, Ruthenium red can still bind to the GalA in non-methylesterified HG and the RG-I backbone. Examination of seeds sections for HG and RG-I epitopes reveals concentrated egg box structures at the outer edge of mucilage, potentially influencing release (**Figure 3**). The distribution of 2F4 epitopes have the same profile of Ruthenium red staining, suggesting that it may substitute calcium ions and fix carboxyl groups in demethylated HG zones, explaining local staining zone. The significance of HG domains becomes apparent when exposed to EDTA treatment, causing disruption to mucilage integrity and releasing fragments. Despite the challenge of understanding EDTA’s rapid impact on mucilage, it emphasizes the significance of localized egg-box structures in mucilage dynamics.

The labeling of JIM7 and JIM5 is also localized in the external and proximal zones, respectively, of the seed surface, which does not entirely correspond with the higher methylation content determined for the adherent mucilage. This could be explained by a higher density of the AM that could alter the recognition of JIM7 epitopes in the inner layer of mucilage. However, to validate this distinctive pattern of HG with varying degrees of methylation, conducting additional labeling with LM19 and LM20, which recognize epitopes distinct from those identified by JIM5 and JIM7, may provide further insights.

Saez-Aguayo et al. (2013) published findings indicating that a mutation in an inhibitor of pectin methylesterase 6 (*PMEI6*) impacts PME activity in the mucilage secretory cell’s periphery, significantly influencing mucilage release. These results subtly suggest that localized alterations in HG structure can have a profound effect on mucilage extrusion, but no direct evidence was described involving HG in mucilage adherence. This observation is interesting since despite the high degree of HG methylesterification in papaya mucilage, its pattern of deposition could influence the mucilage release, as observed in *pmei6* mutant (Saez-Aguayo et al., 2013). The presence of CaCl_2_ enhances wall stiffness, causing strong adhesion of mucilage to the seed surface without release. A similar phenotype is observed in *mum2* mutants in Arabidopsis, where seeds fail to extrude mucilage upon imbibition (Dean et al., 2007). The *mum2* mutation in a β-galactosidase gene leads to elevated Gal side chain in RG-I content compared to the wild type. Additionally, an increase in arabinogalactan type II (AG-II) is also noted, altering mucilage structure, rendering it unable to release under water conditions. These findings emphasize that not only does pectin structure impact mucilage release, but the deposition locus strongly influences pectin release during hydration. Hence, even if egg-box structures are minor in papaya mucilage, their positioning are crucial for maintenance of the bag structure. Utilizing toluidine blue, a cationic dye binding to negatively charged groups (Mitra and Loqué, 2014), dry and hydrated seed slices were stained to elucidate mucilage structure (**Figure 1**). When dry, intense labeling displayed highly compacted mucilage without a columella. Upon hydration, the tegument expanded, and the endotesta disintegrated. Toluidine staining facilitated visualization of two distinct mucilage layers: the soluble and adherent layers.

The use of EDTA facilitates the detachment of the entire mucilage, confirming that demethylesterified HG plays a pivotal role in mucilage adherence, density, and release (**Figure 3**). However, it’s worth noting that this adhesion is not as robust when compared to what is observed in Arabidopsis, particularly in terms of RG-I adhesion on the seed surface.

While GalA is the primary sugar in both mucilage layers, AM has a higher net content of neutral sugars compared to SM. Among all the sugars detected, just GalA and Rha are exclusive for pectins, while the other sugars can be present both in pectins and hemicelluloses. Since AIR is a mixture composed of pectins and hemicelluloses, it is not possible to determine with certainty the presence of other polymers in the mucilage. For better analysis, the AIR from both SM and AM was fractionated into its components: pectins and hemicelluloses (**Supplementary Figure 1**). Based on the AIR analysis it was not unexpected to find that GalA is the main sugar in both SM and AM pectin fractions. In general, the monosaccharide composition of pectins between SM and AM was similar. For instance, the second most abundant sugar in both cases was Xyl, suggesting the presence of xylogalacturonan (Zandleven et al., 2007), xyloglucan (Popper and Fry, 2008; Cornuault et al., 2018) and/or arabinoxylan (Tan et al., 2013). Ara and Gal were also present in high concentrations when considering only the neutral sugars. These monosaccharides are part of RG-I side branches, and considering the low amount of Rha, it is possible to suggest that this pectin domain might be highly branched. The presence of fucose and glucuronic acid could be explained by the presence of arabinogalactan I and II-type side branches of RG-I or traces of xyloglucan contamination that could be identified using antibodies recognizing fucosylated xyloglucan, such as CCRC-M1 (Pattathil et al., 2010; Pattathil et al., 2012; Kaczmarska et al., 2022).

The monosaccharide composition analysis of HC revealed differences between SM and AM. SM was primarily composed of a high proportion of Xyl, followed by Glc, with a marginal amount of Man. Based on these results, it is plausible to propose the presence of three types of HCs: xylan, glucomannan, and xyloglucan. In Arabidopsis mucilage, it has been observed that highly branched xylan is the most abundant Xyl-rich component and is required to maintain mucilage adherence. This is because this branching seems to be necessary for pectin to attach to the seed surface (Voiniciuc et al., 2015b). Additionally, it has also been demonstrated that xylan chains are attached to RG-I chains and mediate the adsorption of mucilage to cellulose microfibrils (Tan et al., 2013; Ralet et al., 2016; Saez-Aguayo et al., 2021). Xyloglucan has also been reported as a seed and root mucilage component (Young et al., 2008; Galloway et al., 2018). In this context, it appears that xyloglucan is more prevalent than xylan in papaya seed mucilage. A dot-blot analysis results using HC from the entire mucilage (SM + AM; **Supplementary Figure 5**) suggest the presence of xylan and traces of heteromannans in the cell wall to some extent. The labeling observed for xyloglucan was considerably more intense, suggesting a higher abundance of these epitope, however, calibration curves must be utilized to quantify and determine the amount of these domains. LM15, which recognizes the XXXG epitope (also recognized by LM25), exhibited the most prominent labeling for xyloglucan. Although LM24’s (XLLG) labeling was faint, the presence of this epitope, even in low abundance, could explain the presence of galactose in the HC fraction, considering both whole mucilage and AM.

Upon analyzing the distribution of LM25 epitopes in mucilage sections, we initially observe a concentrated deposition pattern near the seed surface, confirming the high content of neutral sugars within the hemicellulose fraction of the AM. However, when the mucilage is imbibed, these patterns undergo a transformation, adopting a sheet-like layered configuration in both the AM and SM layers (**Supplementary Figure 6**). This unique deposition form has not been previously observed in mucilage structures. The consistent deposition suggests that these xyloglucan layers contribute to the overall structure but do not seem to play a direct role in mucilage adhesion, especially when the mucilage is exposed to water for an extended period. This observation may help explain the removal of mucilage after 24 h of soaking (**Figure 1**).

On the other hand, the monosaccharide analysis of HC from AM displayed a more diverse range of sugars. Similar to what was observed in SM, the most abundant sugar was Xyl, strongly suggesting the presence of xylan. However, the presence of xyloglucan cannot be ruled out, given that sugars such as Fuc, Ara, Gal and Glc were detected. Their presence could be explained by their potential involvement in substitutions present in xyloglucan (Schultink et al., 2014). Another polysaccharide described specifically for adherent mucilage in Arabidopsis, and whose components are present in the monosaccharide analysis, is galactoglucomannan (Yu et al., 2014). Specifically, it has been documented that Arabidopsis mucilage consists of a highly branched galactoglucomannan rather than an unbranched glucomannan. The extent of galactosylation plays a crucial role in shaping the galactoglucomannan backbone, influencing cellulose structure, mucilage density, and the adhesion of pectin (Voiniciuc et al., 2015c). Therefore, we detected mannan polysaccharides with LM21 in the high-concentration fraction in parallel with biochemical analysis, which determined a higher concentration of heteromannans in the AM fraction. This finding may explain its adherence to the papaya seed surface. Besides primary sugar differences, other monosaccharides may contribute to minor pectin domains like RG-I and RG-II. Utilizing size exclusion chromatography, we isolated specific pectin domains for more tailored approach to analyze their composition. The monosaccharide compositional analysis of OGAs indicates that it is exclusively composed of GalA, as expected for the hydrolysis of a pure fraction of HG domains. However, Xyl is the second most abundant sugar in fractions 41-46 and 47-51, revealing the presence of xylogalacturonan within the papaya mucilage. Notably, in Arabidopsis, xylogalacturonan is present in the root cap mucilage, co-secreted with HG in detached cells from these structures, which serve to shield the roots from soil friction. Recently, there has been a suggestion that XGA could play a role in root defense against pathogens (Durand et al., 2009; Wang and Kang, 2018). Investigating this potential could open promising avenues for future research.

The isolated RG-I domains and their hydrolysis revealed a similar proportion of Rha and GalA that is as expected for this domain. The RG-I branches primarily consist of Ara and Gal. Here the high levels of Ara and Gal measured suggest a highly branched RG-I structure. Furthermore, the presence of Fuc and GlcA suggest the possible presence of AG-I and -II in conjunction with arabinan and galactan. Recent evidence has also shown a covalent linkage between RG-I and AGPs (Tan et al., 2023). AGPs are distinct glycoproteins with large and highly branched polysaccharides attached to via hydroxyproline to a short peptide. In this context, Tan et al. (2023) reported that AG-II, one of the potential RG-I side branches, comprises the carbohydrate component of AGPs and serves as a connecting structure for two RG-I domains. The substantial amount of Xyl measured in the fraction could be explained by the presence of arabinoxylan 1, which can be bound to the AG-II side branches, or by arabinoxylan 2, which can be directly linked to a Rha residue in the RG-I backbone (Tan et al., 2013). However, contamination by HC could not be excluded. The monosaccharide composition analysis was complemented by dot-blot using antibodies recognizing RG-I epitopes. INRA-RU1, INRA-RU2, LM6, LM26, LM25 and LM30 antibodies yielded positive results with varied in intensity (**Supplementary Figure 4**). Both INRA-RU1 and INRA-RU2 produced a similar spot for the highest amount of RG-I loaded onto the membrane. These antibodies recognize unbranched RG-I suggesting that most of the RG-I domain is not branched. This aligns with the high amount of Ara and Gal measured. LM6 also exhibited a spot, this antibody recognizes (1-5)-α-L-arabinans present in the arabinan side chains, as well as in AG-I and -II. LM30 recognizes the arabinosyl residues of AGP. Its label is slightly fainter than LM6, and both antibodies have a similar pattern. This suggests that the isolated RG-I may have AGP attached, and some of the detected Ara may be part of AG-II. Finally, LM26 presented a very faint spot. This antibody recognizes β-1,6-galactosyl substitutions of β-1,4-galactan, requiring more than three backbone residues; it has no recognition of linear (1-4)-β-D-galactan (Torode et al., 2018). The faint label for this antibody suggests that the galactan present in RG-I may be linear without substitutions. Numerous indications point to the role of galactan-rich RG-I in enhancing the rigidity of plant tissues (McCartney et al., 2000), whereas arabinans have been linked to the flexibility of guard cells (Jones et al., 2003). Moreover, side chains abundant in arabinans and galactans exhibit high mobility and are likely to possess water-holding capabilities (Ha et al., 2005). An important aspect to consider is the outcome of antibodies LM26 and LM30 in seed cross-sections. Both antibodies are located very close to the seed coat, displaying a similar distribution and behavior in both dry and hydrated seeds. This result could indicate that, in a way, both antibodies are recognizing the same component. LM26 recognizes (1,4)-galactan, which is present, for example, in AG-II, which can also serve as a side chain of RG-I and be part of AGPs. LM30, in turn, recognizes AGPs, which may also be connected to RG-I (Tan et al., 2023). Based on this result, it could be suggested that there is a connection between RG-I and AGPs, which could be involved in anchoring the mucilage to the seed surface of papaya.

Given the quantity of Xyl and Glc detected in the isolated RG-I fraction, which are primarily components of hemicellulosic polysaccharides, we decided to analyze the origin of their presence in this fraction. We can rule out the presence of xylan due to the negative reaction observed with LM10 and LM11 antibodies. Therefore, we analyzed the presence of xyloglucan using the LM25 antibody. The detection of an intense label in LM25 strongly suggests the presence of xyloglucan, a conclusion supported by the presence of Xyl and Glc in the acid hydrolysis. This leads us to propose that xyloglucan might directly bind to RG-I, forming a network that contributes to the maintenance of the mucilage structure and stiffness. Finally, the latest minor pectin domain analyzed was RG-II, and it is arguably the most challenging domain to analyze due to its complex structure, which comprises 13 sugars and 21 glycosidic linkages organized into six side branches of a GalA backbone (Ndhe et al., 2017). RG-II contains some very low-abundance sugars such as apiose, 3-deoxy-d-manno-oct-2-ulosonic acid (Kdo), and aceric acid that cannot be detected using our HPLC conditions. Therefore, we opted to analyze this domain using PAGE, which allows the separation of the dimeric and monomeric forms of RG-II (Chormova et al., 2014). The results indicate that RG-II is predominantly in its dimeric form, with an almost negligible band at the monomeric size (**Supplementary Figure 3**). These results provide evidence for the presence of RG-II in both mucilage layers of papaya, implying the involvement of this domain in mucilage structure and cohesion. Nevertheless, further analysis must be conducted to ascertain the significance of this minor domain in the overall mucilage network structure.

## 5 Conclusions

**Figure 8:**
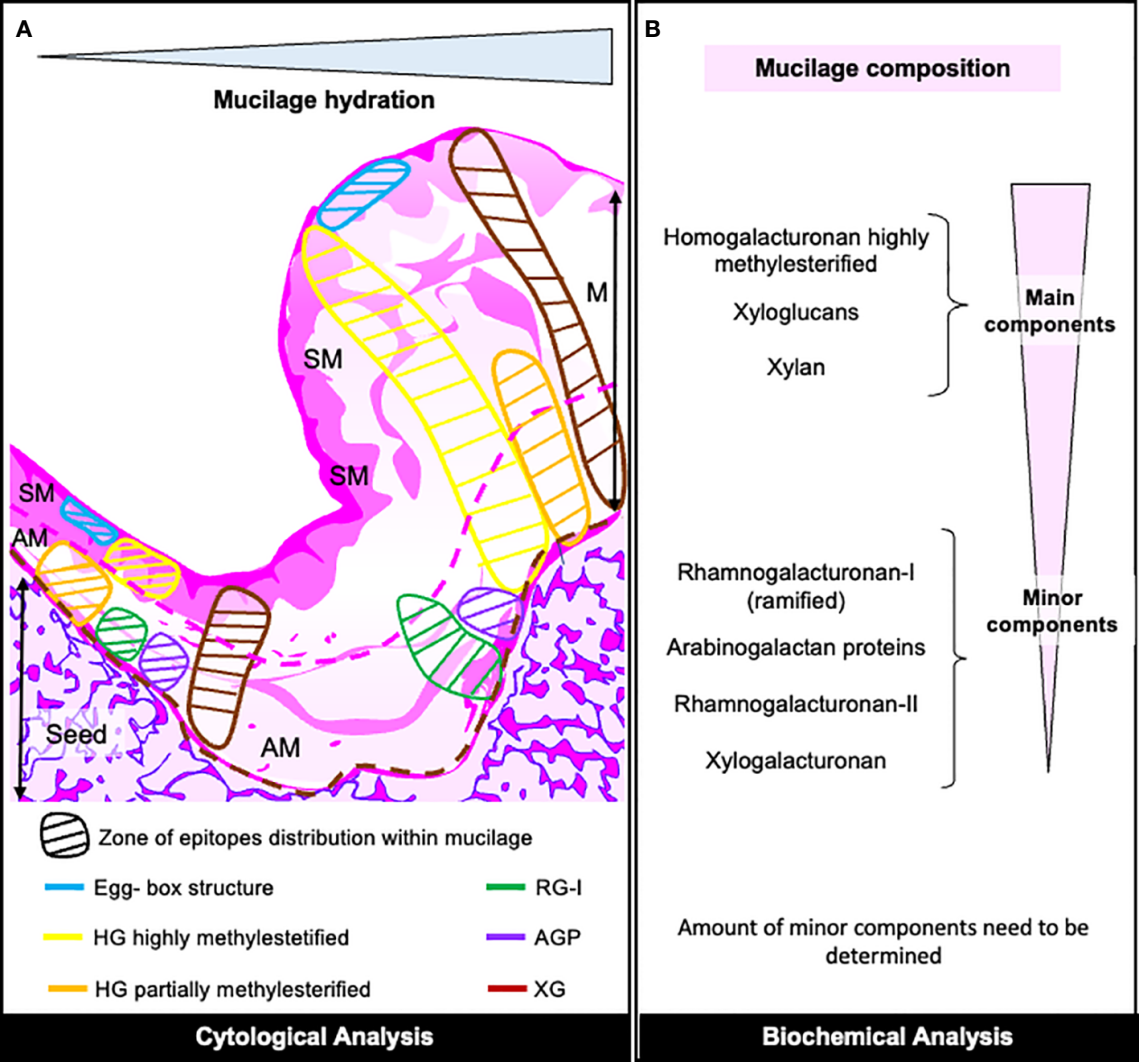
Overview of Papaya Mucilage Composition. **(A)** Diagram illustrating the distribution pattern of mucilage epitopes as determined by cytological analysis within the mucilage section. The hypothetical limits of AM (adherent mucilage) and SM (seed mucilage) are represented by pink-red lines, while the brown dotted line delineates the seed surface. HG, homogalacturonan; RG-I, rhamnogalacturonan-I; AGP, arabinogalactan protein; XG, xyloglucan. **(B)** Description of mucilage components in both mucilage layers, determined through various biochemical analyses. The quantities of minor components require further verification.

With the strategy developed in this research, we have characterized the composition of papaya mucilages, which are primarily composed of HG and xyloglucans (as shown in **Figure 8**). Additionally, it contains a significant amount of xylan and traces of RG-I, RG-II, XGA, mannans and AGPs. The purification and analysis of the RG-I domain reveal that this minor pectic domain is branched with AGPs and Xyloglucans, implying its involvement in mucilage adherence. The adhesion of papaya mucilage is relatively short-lived, given that its main component is HG, and it does not exhibit strong adhesion with HC and cellulose, in contrast to RG-I as studied in Arabidopsis. Furthermore, we have detected traces of XGA and RG-II in the entire mucilage, suggesting their role in mucilage structure. This novel characterization of mucilage raises questions about the potential utilization of pectin derived from discarded fruit in various industrial applications.

## Data availability statement

The datasets presented in this study can be found in online repositories. The names of the repository/repositories and accession number(s) can be found in the article/**Supplementary Material**.

## Author contributions

DS: Conceptualization, Data curation, Formal analysis, Investigation, Methodology, Writing – original draft, Writing – review & editing. PS-O: Investigation, Methodology, Writing – original draft. AS: Methodology, Writing – original draft. SZ: Methodology, Writing – original draft. RH: Writing – review & editing, Funding acquisition. MM-L: Writing – review & editing, Funding acquisition. SS: Conceptualization, Data curation, Formal analysis, Funding acquisition, Investigation, Methodology, Resources, Supervision, Writing – original draft, Writing – review & editing.

## Supplementary material

Supplementary Figure 1 Schematic representation of papaya mucilage extraction procedure used for sugar analyses. M, mucilage; AM, adherent mucilage; DS, demucilaged seed; WM, whole mucilage.

Supplementary Figure 2 Sugar composition of papaya seed mucilage layers. **(A)** Schematic representation of papaya mucilage. SM, soluble mucilage; AM, adherent mucilage; DS, demucilaged seed. **(B)** Monosaccharide composition of each papaya mucilage layer determined by HPAEC after TFA hydrolysis. Values represent means and SE from 3 biological replicates (n = 12) and are expressed as mg/g of AIR.

Supplementary Figure 3 The presence of rhamnogalacturonan-II in soluble mucilage (SM) and adherent mucilage (AM) was determined. The AIR of both mucilage types was digested with endoPG and subjected to PAGE analysis. The resulting bands were stained with silver. Each lane on the gel represents 8 μL of a supernatant obtained from the digested AIR, with a concentration of 1 mg/μL. M, monomer, D, dimer.

Supplementary Figure 4 Dot-blot isolated RG-I domain. The isolated RG-I domain was subjected to analysis for the presence of specific epitopes using several antibodies: INRA-RU1 and INRA-RU2 recognize the unbranched RG-I backbone, LM26 (anti-galactan), LM6 (anti-arabinan), LM30 (anti-AGPs), LM10 and LM11 (anti-xylan), and LM25 (anti-xyloglucan, XXXG, XLLG, XXLG). A volume of 0.7 µL from various dilutions was spotted onto a nitrocellulose membrane.

Supplementary Figure 5 Dot-blot analysis of the hemicellulose fraction. As a loading sample we used total HC, SM + AM. We selected two antibodies, CCR-M139 and LM10, which both showed a faint label, indicating the low abundance of this polysaccharide. However, they exhibited a similar profile in terms of intensity and label distribution. For xyloglucan analysis, we selected three antibodies: LM15 (XXXG), LM24 (XLLG), and LM25 (XLLG, XXLG, and XXXG). LM15 and LM25 exhibited similar profiles, with LM15 showing slightly stronger labeling, specifically recognizing the XXXG epitope. This suggests that the labeling observed for LM25 is likely due to the presence of the same epitope, especially considering that LM24 produced only a mild label. Additionally, the LM21 antibody allows for the observation of the presence of heteromannan through the β-1,4-mannan epitope.

Supplementary Figure 6
*In situ* xyloglucan distribution in papaya mucilage determined by immunofluorescence. Immunofluorescence analyses were performed on dehydrated seeds and water-soaked papaya mucilage using the LM25 antibody labeling in magenta and Calcofluor white staining for glycans and seed coat in gray. SC, seed coat; M, mucilage. Scale bar: 30 µm.

Supplementary Table 1 Antibodies used in Immunodot Blot assays of papaya mucilage fraction polysaccharides.
