# Supplementary material for: Mucilage extracted from Chilean papaya seeds is enriched with homogalacturonan domains
Source: Front Plant Sci. 2024 May 30;15:1380533. doi: 10.3389/fpls.2024.1380533 (PMC11169631; doi:10.3389/fpls.2024.1380533)
Supplement: Supplementary Figure 1 — Schematic representation of papaya mucilage extraction procedure used for sugar analyses. M, mucilage; AM, adherent mucilage; DS, demucilaged seed; WM, whole mucilage. [file Presentation_1.pdf]

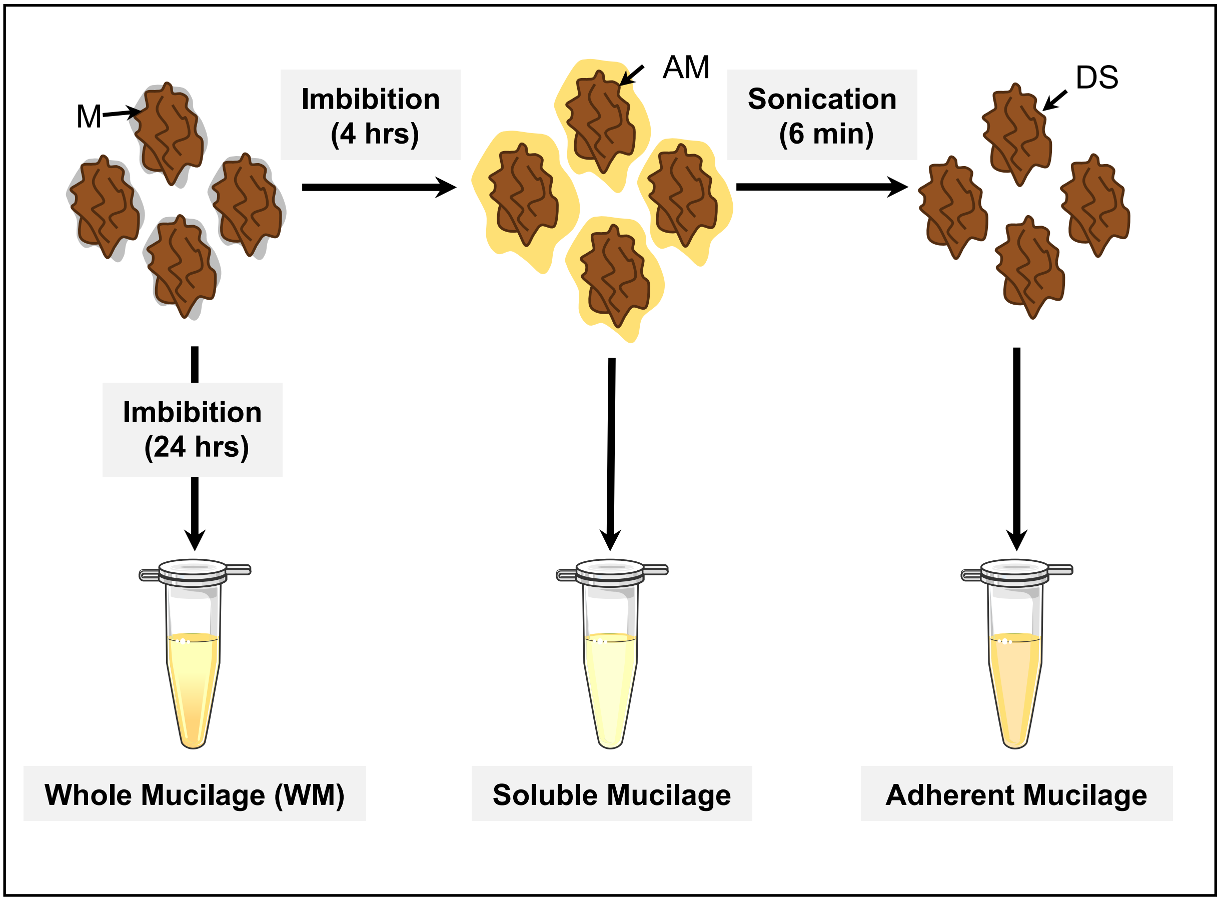

Supplementary Figure 1: Schematic representation of papaya mucilage extraction procedure used for sugar analyses. M, mucilage; AM, adherent mucilage; DS, demucilaged seed; WM, whole mucilage

A Papaya mucilage structure scheme

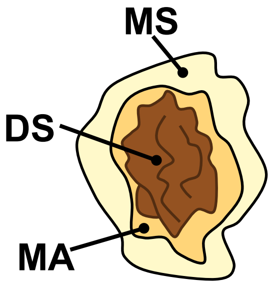

B

	Sugar Composition in mg/g of AIR	
Sugar type	SM	AM
Rha	19.0 (0.47)	30.2 (3.39)
Ara	16.7 (1.04)	24.5 (2.21)
Gal	28.2 (0.77)	44.7 (2.44)
Glc	22.8 (1.19)	31.2 (1.13)
Man	10.2 (0.33)	19.0 (1.56)
Xyl	28.6 (1.00)	66.0 (5.98)
Fuc	2.6 (0.17)	8.1 (0.84)
GalA	325.0 (29.32)	231.6 (11.33)
GlcA	6.6 (0.38)	4.5 (0.29)
Total Sugars	459.8 (29.67)	459.8 (27.73)

Supplementary Figure 2: Sugar composition of papaya seed mucilage layers.

(A) Schematic representation of papaya mucilage. SM, soluble mucilage; AM, adherent mucilage; DS, demucilaged seed. (B) Monosaccharide composition of each papaya mucilage layer determined by HPAEC after TFA hydrolysis. Values represent means and SE from 3 biological replicates (n = 12) and are expressed as mg/g of AIR.

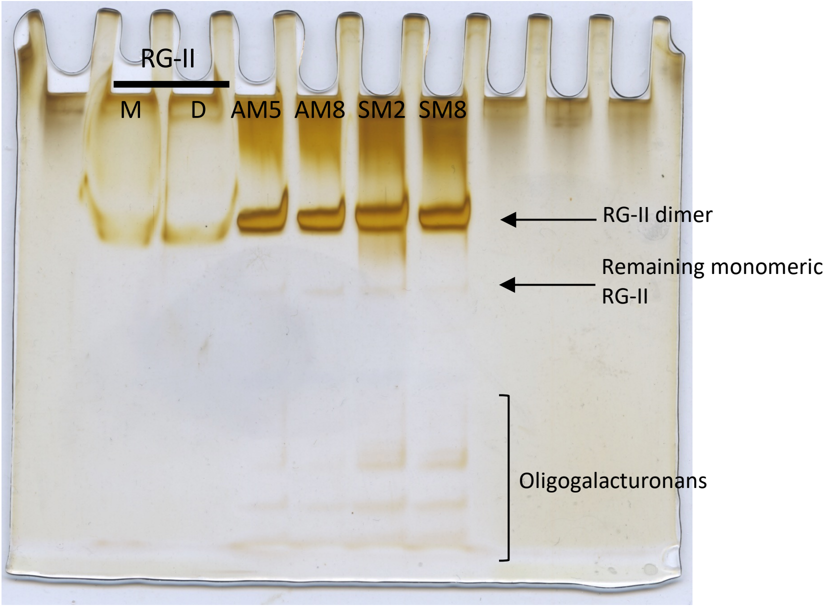

Supplementary Figure 3: The presence of rhamnogalacturonan-II in soluble mucilage (SM) and adherent mucilage (AM) was determined. The AIR of both mucilage types was digested with endoPG and subjected to PAGE analysis. The resulting bands were stained with silver. Each lane on the gel represents 8 µL of a supernatant obtained from the digested AIR, with a concentration of 1 mg/µL. M, monomer, D, dimer.

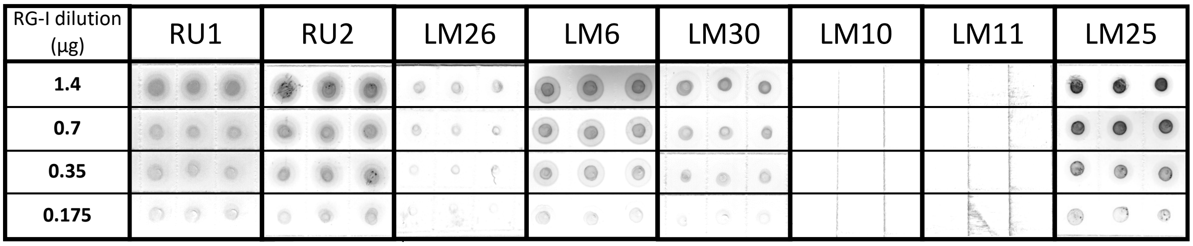

Supplementary Figure 4: Dot-blot isolated RG-I domain.

The isolated RG-I domain was subjected to analysis for the presence of specific epitopes using several antibodies: INRA-RU1 and INRA-RU2 recognize the unbranched RG-I backbone, LM26 (anti-galactan), LM6 (anti-arabinan), LM30 (anti-AGPs), LM10 and LM11 (anti-xylan), and LM25 (anti-xyloglucan, XXXG, XLLG, XXLG). A volume of 0.7 µL from various dilutions was spotted onto a nitrocellulose membrane.

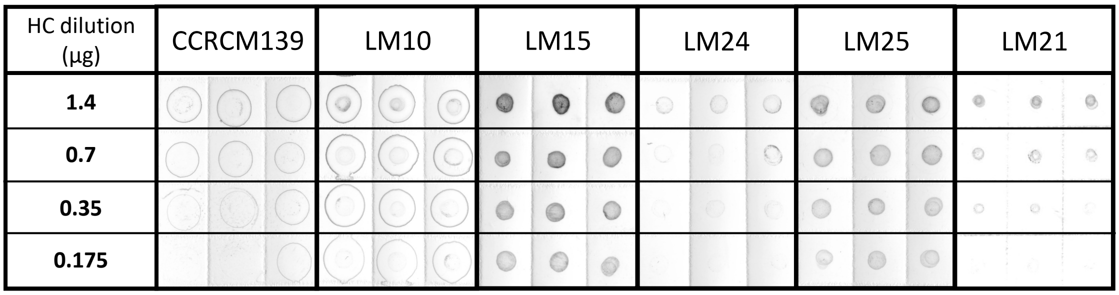

Supplementary Figure 5: Dot-blot analysis of the hemicellulose fraction.

As a loading sample we used total HC, SM + AM. We selected two antibodies, CCR-M139 and LM10, which both showed a faint label, indicating the low abundance of this polysaccharide. However, they exhibited a similar profile in terms of intensity and label distribution. For xyloglucan analysis, we selected three antibodies: LM15 (XXXG), LM24 (XLLG), and LM25 (XLLG, XXLG, and XXXG). LM15 and LM25 exhibited similar profiles, with LM15 showing slightly stronger labeling, specifically recognizing the XXXG epitope. This suggests that the labeling observed for LM25 is likely due to the presence of the same epitope, especially considering that LM24 produced only a mild label. Additionally, the LM21 antibody allows for the observation of the presence of heteromannan through the β-1,4-mannan epitope.

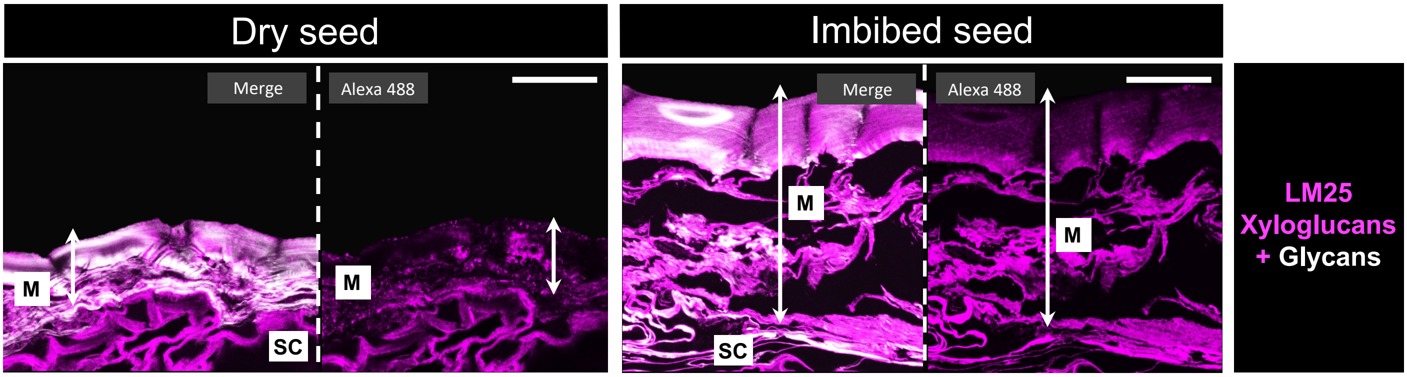

Supplementary Figure 6 : *In situ* xyloglucans distribution in papaya mucilage determined by immunofluorescence

Immunofluorescence analyses were performed on dehydrated seeds and water-soaked papaya mucilage using the LM25 antibody labeling in magenta and Calcofluor white staining for glycans and seed coat in gray . SC, seed coat; M, mucilage. Scale bar: 30 µm.

Antibody	Specificity	Category	Ab dilution	Reference
**Pectins**				
INRA-RU1	Anti-unbranched RG-I	Monoclonal	01:50	Ralet et at., 2010
INRA-RU2	Anti-unbranched RG-I	Monoclonal	01:50	Ralet et at., 2010
LM6	Anti-arabinan	Monoclonal	01:50	Willats et al., 1998
LM26	Anti-branched galactan	Monoclonal	01:50	Torode et al., 2018
**Hemicelluloses**				
LM10	Anti-unsubstituted and low-substituted xylans	Monoclonal	01:50	McCartney et al., 2005; Ruprecht et al., 2017
LM11	Anti-xylan/arabinoxylan	Monoclonal	01:50	McCartney et al., 2005; Ruprecht et al., 2017
LM21	Anti-mannan	Monoclonal	01:50	Marcus et al., 2010
LM15	Anti-xyloglucan	Monoclonal	01:50	Marcus et al., 2008 Moller et al., 2008
LM24	Anti-xyloglucan	Monoclonal	01:50	Pedersen et al., 2012
LM25	Anti-xyloglucan	Monoclonal	01:50	Pedersen et al., 2012
CCRC-M139	Anti-xylan	Monoclonal	01:50	Pattathil et al., 2010
**Proteoglycan**				
LM30	Anti-arabinogalactan protein	Monoclonal	01:50	Moller et al., 2008

Supplementary Table 1: Antibodies used in Immunodot Blot assays of papaya mucilage fraction polysaccharides

Supplementary References:

Marcus, S. E., Verhertbruggen, Y., Hervé, C., Ordaz-Ortiz, J. J., Farkas, V., Pedersen, H. L., et al. (2008). Pectic homogalacturonan masks abundant sets of xyloglucan epitopes in plant cell walls. BMC plant biology, 8, 1-12. doi: doi.org/10.1186/1471-2229-8-60

Marcus, S. E., Blake, A. W., Benians, T. A., Lee, K. J., Poyser, C., Donaldson, L., et al. (2010). Restricted access of proteins to mannan polysaccharides in intact plant cell walls. The Plant Journal, 64(2), 191-203. doi: 10.1111/j.1365-313X.2010.04319.x
